# Proof of concept pilot study to assess the utility of magnetic resonance extra-cellular volume quantification to diagnose advanced liver disease in people with Cystic Fibrosis

**DOI:** 10.1371/journal.pone.0318085

**Published:** 2025-03-04

**Authors:** Daniel H. Tewkesbury, Jennifer A. Scott, Rowland J. Bright-Thomas, Sue Liong, Josephine Naish, Velauthan Rudralingam, Karen Piper Hanley, Andrew M. Jones, Varinder S. Athwal

**Affiliations:** 1 Manchester University NHS Foundation Trust, Manchester, United Kingdom; 2 Division of Immunology, Immunity to Infection and Respiratory Medicine, Faculty of Biology, Medicine and Health, University of Manchester, Manchester, United Kingdom; 3 Division of Diabetes, Endocrine and Gastroenterology, Faculty of Biology, Medicine and Health, University of Manchester, Manchester, United Kingdom; Linköping University, SWEDEN

## Abstract

**Background:**

Current diagnostic tools are limited in their ability to diagnose cystic fibrosis liver disease (CFLD) as disease is often focal in nature. Magnetic resonance extracellular volume quantification (MRI ECV) in the liver may have diagnostic utility in CFLD as a more selective liver volume is assessed and can be performed using equipment readily available in clinical practice on a standard MRI protocol.

**Methods:**

Healthy volunteers (HV), CF participants with no liver disease (CF-noLD) and CF participants with cirrhosis (CF-C) aged 18 years and above had MRI ECV measured using a 3T Siemens scanner. An additional retrospective analysis was performed to calculate MRI ECV in individuals who had available images obtained using a 1.5T Siemens scanner from a previous study.

**Results:**

16 individuals had MRI ECV measured using a 3T Siemens scanner. Mean (SD) MRI ECV was 0.316 (0.058) for HV (n  =  5), 0.297 (0.034) for CF-noLD (n  =  5) and 0.388 (0.067) for CF-C (n  = 6 ). Post-hoc analysis showed a significant difference between CF-noLD and CF-C (*p*  =  0.046). Of 18 individuals with available images using a 1.5T scanner, mean (SD) MRI ECV was 0.269 (0.048) in HV (n  =  8), 0.310 (0.037) in CF-noLD (n  =  8) and 0.362 (0.063) in CF-C (n  =  2).

**Conclusions:**

Liver MRI ECV quantification was feasible in adults with CF with no significant difference in results between 1.5T and 3T obtained images suggesting applicability across different types of MRI scanner. A higher MRI ECV was demonstrated in CF participants with cirrhosis suggesting potential utility as a diagnostic tool for those with advanced CFLD. Further evaluation in larger cohorts is warranted.

## Introduction

Cystic fibrosis (CF) is the most common lethal inherited condition in the Western world [1]. Mutations to the cystic fibrosis transmembrane conductance regulator (*CFTR*) gene result in abnormal chloride and bicarbonate transport across epithelial cells, causing multisystem disease. In addition to common features such as bronchiectasis and exocrine pancreatic insufficiency, some people with cystic fibrosis (PwCF) develop a form of chronic liver disease known as cystic fibrosis related liver disease (CFLD), with a reported prevalence of up to 32% in a large French cohort study [2].

Although the precise aetiology remains unclear [3], development of CFLD is associated with increased risk of mortality [4] and is the most common non-respiratory cause of death in PwCF [5]. Thanks to improvements in nutritional and respiratory management of PwCF and the development of therapies which restore function to the defective CFTR protein (CFTR modulators), life expectancy has increased over recent decades. It is currently unknown how increased life expectancy or restoration of CFTR function for those on CFTR modulator therapies will affect the prevalence of CFLD, though some studies have shown that incidence of CFLD continues throughout adulthood, highlighting the need for ongoing CFLD screening in adults with CF [2,6].

Clinical features of CFLD are heterogeneous and often subclinical, meaning that advanced disease is often underdiagnosed [2]. The CF Foundation currently classifies CFLD into the following categories [7]:

- Liver involvement with cirrhosis or portal hypertension (PHT).
- Liver involvement without cirrhosis or PHT.
- No evidence of liver involvement.

Liver fibrosis in CFLD is often focal in nature, making liver biopsy (the usual diagnostic gold-standard in other forms of chronic liver disease) unreliable. Non-invasive measures of liver stiffness such as FibroScan™ have demonstrated an ability to determine the presence of advanced CFLD [8], although these techniques may be limited in their ability to detect early disease [9,10]. Moreover, elastography can be unreliable in the presence of obesity or ascites [11] and there is concern that reliability for CFLD diagnosis may be limited given the heterogeneous distribution of fibrosis that is characteristic of advanced CFLD [12], as a volume of only 3 cm^3^ in the right lobe is assessed [13].

Magnetic resonance imaging (MRI) can identify structural complications of CFLD-cirrhosis, however MRI techniques needed to diagnose earlier stages of fibrosis, such as MR elastography (MRE), require additional expensive equipment and technology which is not readily available in clinical practice and may be affected by factors such as inflammation, steatosis and hepatic venous congestion [14].

In contrast, calculation of MRI ECV (extra cellular volume) can be performed in routine clinical settings, using a standard MRI protocol with no additional equipment [15,16], and is clinically established in cardiology practice to detect cardiac fibrosis following myocardial infarction [17]. This technique calculates ECV by comparing concentrations of contrast agent in the extracellular space (which expands in fibrosis) with the blood in a dynamic steady state [18]. Compared to other MRI techniques, contrast-enhanced ECV calculation is less affected by steatosis, iron content, oedema and field strength [19,20].

There is early evidence to suggest that MRI ECV in the liver can be used as a marker for liver fibrosis. Liver MRI ECV detected an increase in scar tissue in mice with liver fibrosis, which correlated with collagen staining [21]. In addition, patients with chronic hepatitis B had strong correlation between MRI ECV levels and histology [22]. In a patchy and focal disease such as CFLD, MRI ECV could potentially measure liver fibrosis more selectively and in different areas.

The aim of this pilot study is to assess the feasibility of using MRI ECV to detect liver fibrosis in CF adults with advanced CFLD.

## Methods

### Participants

Participants were recruited into one of three groups: healthy controls, CF with no liver disease, and CF with established cirrhosis.

Participants in all groups had to meet the following eligibility criteria: age over 18 years, no contraindication to MRI scan, no recorded hypersensitivity to a contrast agent, estimated glomerular filtration rate (eGFR) > 50 ml/min/1.73m^2^. PwCF with implanted venous access ports were not included as MRI compatibility of devices could not be established in these individuals.

CF participants were recruited from Manchester Adult Cystic Fibrosis Centre from 28/02/2019 to 21/06/2023 with all participants providing informed written consent. CFLD was defined according to established clinical (Debray) criteria of two or more of the following [23]:

- Abnormal physical examination including hepatomegaly or splenomegaly
- Abnormalities of liver function tests defined as an increase in transaminases or gamma-glutamyl transferase levels above the upper limit of normal on at least 3 consecutive determinations over 12 months after excluding other causes of liver disease
- Ultrasonographic evidence of liver involvement (increased and/or heterogeneous echogenicity, irregular margins, nodularity), portal hypertension, or biliary abnormalities (bile duct dilatation)
- Histological evidence of CFLD if a liver biopsy has been performed

Given the lack of diagnostic accuracy, PwCF do not routinely undergo liver biopsies in clinical practice. The presence of cirrhosis was defined as the presence of one of the following on recent imaging (within the previous 2 years): a small, shrunken liver; caudate hypertrophy, liver contour nodularity or evidence of portal hypertension.

### Equipment and MRI protocol

MRI scans were performed on a 3T Siemens Magnetom Vida scanner (Siemens Healthcare, Erlangen, Germany), using a matrix body coil. The MRI protocol lasted approximately 45 minutes and consisted of initial abdominal and cardiac localiser scans, followed by a structural T2w Half fourier Single-shot Turbo spin-Echo (HASTE) with multi breath hold scan and a T1w multi echo volumetric interpolated breath-hold examination (VIBE) Dixon (1.34/4.20 TE/TR, slice thickness 3mm with 20% distance).

Pre-contrast axial T1 maps of the liver were acquired using a 5(3)3 MOLLI acquisition (TE/TR 1.03/263.4, 5x8mm slices, distance factor 20%, FOV  =  440x330mm, matrix 156x192). A T1 map of the heart was also acquired (single slice, mid short axis) to obtain blood pool T1. T1 maps were generated inline using Siemens MyoMaps software. Gadoterate meglumine (Doterem) contrast agent was administered as a bolus at a dose of 0.15mmol/kg of body-weight and T1 mapping was repeated in the liver and heart 15 minutes post contrast using a 4(1)3(1)2 MOLLI acquisition. Prior to post-contrast T1-mapping, a second post-contrast T_1_w VIBE Dixon was acquired.

### Calculation of ECV

Liver ECV was calculated using T1 relaxation times in liver parenchyma and in blood in a dynamic steady state. It was calculated using the following equation:


$$\text{ECV=}\left(\text{1-heamatocrit}\right)\text{x }\left({\text{ΔR1}}_{\text{tissue}}{\text{/ΔR1}}_{\text{blood}}\right)$$

$$\text{ΔR1=}{\left(\text{1/T1}\right)}_{\text{equib}}\text{–}{\left(\text{1/T1}\right)}_{\text{pre}}$$

(ΔR1 =  change in relaxation rate)

**Fig 1:**
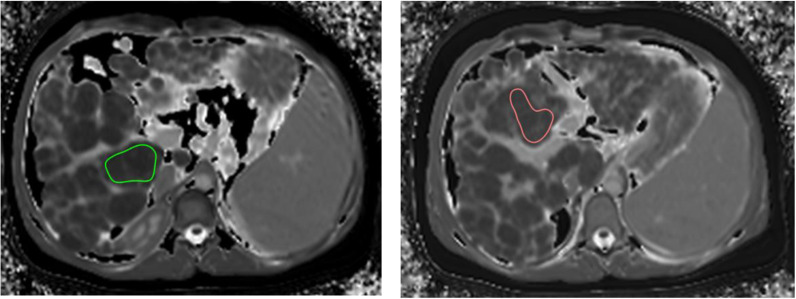
Example of regions of interest used to measure T1 relaxation times in liver parenchyma. Areas of regions of interest: 9.47 cm^2^ (left hand image), 8.07 cm^2^ (right hand image). Slice thickness 0.8 cm.

DICOM software (Horos) was used to measure T1 relaxation times from tissue (liver parenchyma) and blood in order to calculate ECV according to the above equation. T1 relaxation times in the blood were measured in the left ventricle of the heart during diastole. In the liver T1 relaxation times were measured by drawing two regions of interest (ROI) in different lobes of the liver (2 cm away from the liver edge and avoiding any large vessels) and taking an average. The largest available contiguous areas of liver parenchyma in different lobes of the liver were selected. Where applicable, ROIs could be selectively drawn in areas displaying macronodular change and this was done by selecting the largest available contiguous areas of nodular liver (see Fig 1). DICOM software allowed for measurement of post-contrast T1 relaxation times in the same locations (using a ‘copy and paste’ function) at 15 minutes following the administration of intravenous gadolinium-based contrast. Increasing the number of regions of interest (up to 5 ROIs tested) did not make a significant difference to mean values. As such, 2 ROIs were chosen as a parsimonious solution with clinical ease of use.

### Liver stiffness measurement

On the same day as MRI scan, participants had an assessment of liver stiffness using a FibroScan Touch 502 (Echosens, Paris, France) by a fully trained operator. Controlled attenuation parameter (CAP) is a further measurement produced by FibroScan which has shown to correlate with hepatic steatosis [24]. The measurement ranges from 100-400 dB/m with a value > 260 dB/m regarded as moderate steatosis [24].

### Retrospective data from a 1.5T Siemens scanner

To assess the feasibility of MRI ECV using a different type of scanner more commonly used in clinical practice, a retrospective review was performed on an additional cohort of individuals from a previous study at our institution (covered by the same ethical approval). This included healthy controls, CF individuals with no CFLD and CF individuals with CFLD-cirrhosis according to the criteria above.

These individuals had an MRI scan on a 1.5 Tesla (T) scanner (Magnetom Avanto, Siemens Healthcare Secor, Erlangen, Germany) equipped with a 32-element phased-array coil. The protocol of these scans was similar to studies performed by Miller et al. and used a standard gadolinium-based contrast agent [25]. MRI ECV was calculated using the same method described above.

### Statistical analysis

Baseline data are presented as mean and standard deviation (SD). A one-way ANOVA with post-hoc Tukey’s was used to analyse differences in mean MRI ECV between groups. Statistical analysis was performed using GraphPad Prism version 9.

### Ethical approval

This study received ethical approval from the Health Research Authority Research Ethics Committee (North West – Greater Manchester West; reference 18/NW/0827).

## Results

### Demographics

A total of 16 participants were recruited including 5 healthy controls, 5 CF participants with no liver disease and 6 CF participants with CFLD cirrhosis. One participant in the healthy control group had to attend twice for an MRI scan due to a fault with the equipment on the first visit. Demographic information is presented in Table 1 including body mass index (BMI) which was calculated from weight and height measurements taken on the day. Value of percentage predicted forced expiratory volume in 1 second (ppFEV_1_) for CF participants was taken from their most recent clinic assessment within 3 months of MRI scan date.

**Table 1:** Demographic information.

		Healthy controls (n = 5)	CF with no liver disease (n = 5)	CF with cirrhosis (n = 6)
**Age (SD)**		30 (1.3)	34 (7.5)	24 (10.5)
**Sex (%)**	*Male*	3 (60%)	4 (80%)	4 (66.7%)
**Ethnicity (%)**	*Caucasian*	5 (100%)	5 (100%)	6 (100%)
**BMI (SD)**		24.8 (1.5)	26.8 (2.4)	23.0 (3.7)
**Liver stiffness measurement, kPa (SD)**		4.2 (0.9)	4.6 (0.9)	12 (5.8)
**Controlled attenuation parameter, dB/m (SD)**		157 (38)	234 (80)	223 (87)
**ppFEV**_1_ **(SD)**		–	80.0 (9.7)	79.5 (12.7)
**Exocrine pancreas insufficiency (%)**	*Present*	–	4 (80%)	6 (100%)
**CFRD (%)**	*Present*	–	1 (20%)	3 (50%)
**CFTR modulator treatment**	*E/T/I*	–	5 (100%)	6 (100%)

BMI: body mass index; CF: cystic fibrosis; CFRD: cystic fibrosis related diabetes; CFTR: cystic fibrosis transmembrane conductance regulator; E/T/I: elexacaftor/tezacaftor/ivacaftor; ppFEV_1_: percentage predicted forced expiratory volume in 1 second; SD: standard deviation

### MRI ECV differences between groups

**Fig 2:**
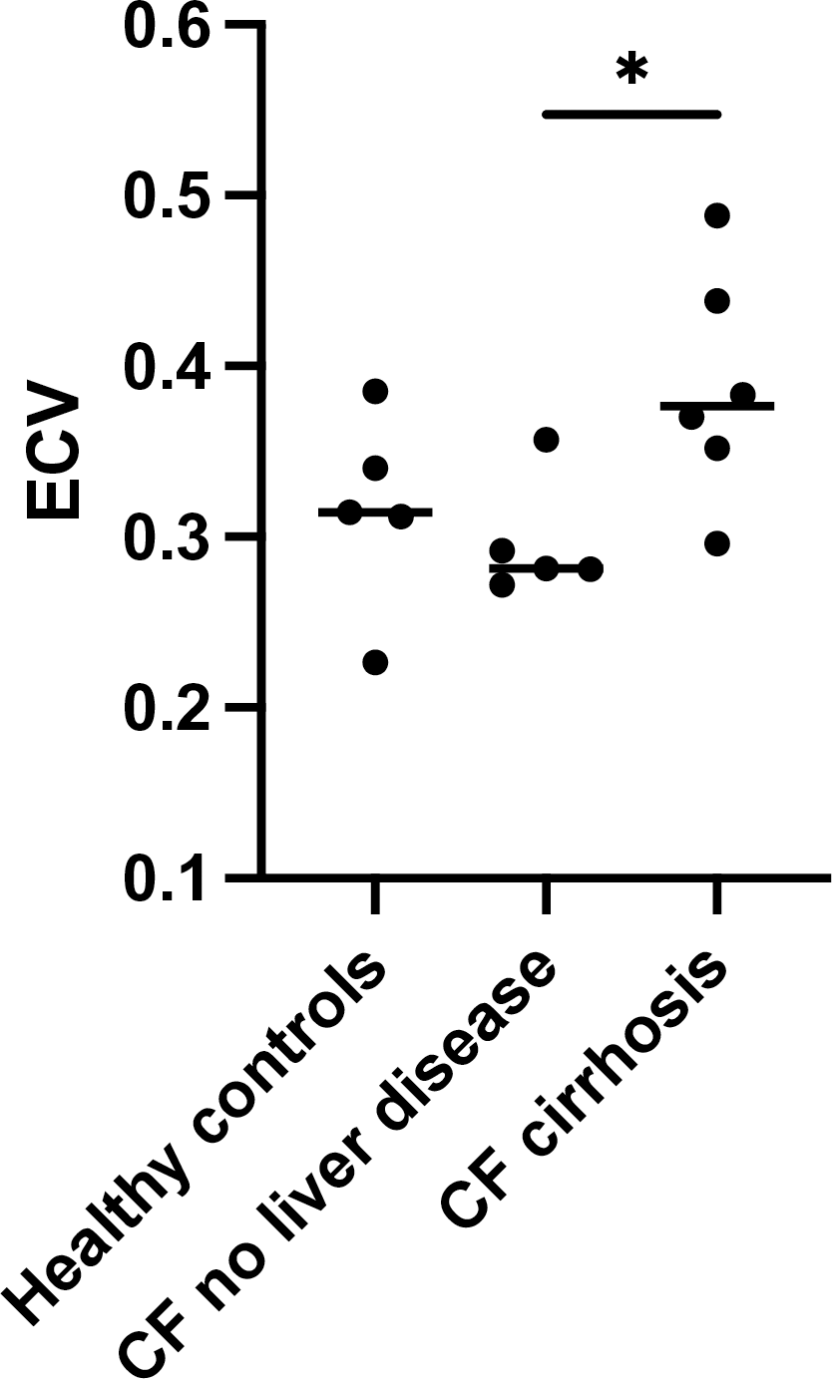
MRI ECV values according to group on 3T MRI scanner. MRI Liver ECV calculated from a 1.5T Siemens scanner.

Mean liver ECV in the healthy controls (0.316, SD 0.058) was not significantly different compared to mean liver ECV in CF participants without liver disease (0.297, SD 0.034) *p* =  0.55. Liver ECV in healthy controls was lower compared to mean liver ECV in those with CF and cirrhosis (0.388, SD 0.067), however this did not reach statistical significance (*p* =  0.09). Mean liver ECV was in CF participants with no liver disease (0.297, SD 0.034) was significantly lower compared to those with CF and cirrhosis (0.388, SD 0.067), *p* =  0.046. The distribution of results in each group is shown in Fig 2.

Data from 18 individuals who underwent MRI using a 1.5T Siemens scanner was analysed, including 8 healthy volunteers, 8 CF participants with no liver disease, and 2 CF participants with cirrhosis. Demographic data was reviewed retrospectively (Table 2) though some information including ethnicity was not available. 4 CF participants with no liver disease and 1 CF participant with cirrhosis had a recent (within 3 months of MRI scan date) assessment of liver stiffness using FibroScan.

**Table 2:** Demographic data of 10 patients with CF from 1.5T Siemens MRI.

		Healthy controls (n = 8)	CF with no liver disease (n = 8)	CF with cirrhosis (n = 2)
**Age (SD)**		46 (8.7)	32 (7.6)	30 (10.6)
**Sex (%)**	*Male*	–	6 (75%)	2 (100%)
**BMI (SD)**		–	23.3 (3.4)	24.2 (3.5)
**Liver stiffness measurement, kPa (SD)** ^a^		–	4.1 (0.9)	11.8
**Controlled attenuation parameter, dB/m (SD)** ^a^		–	249 (87)	228
**ppFEV**_1_ **(SD)**		–	53.8 (11.8)	63.2 (4)
**Exocrine pancreas insufficiency (%)**	*Present*	–	8 (100%)	2 (100%)
**CFRD (%)**	*Present*	–	4 (50%)	2 (100%)
**CFTR modulator treatment**	*LUM/IVA*	–	3 (38%)	0 (0%)

BMI: body mass index; CF: cystic fibrosis; CFRD: cystic fibrosis related diabetes; CFTR: cystic fibrosis transmembrane conductance regulator; LUM/IVA: lumacaftor/ivacaftor; ppFEV_1_: percentage predicted forced expiratory volume in 1 second; SD: standard deviation.

^a^Only 4 CF control patients and 1 CF with cirrhosis patient had FibroScan results.

Mean MRI ECV values for healthy volunteers, CF participants with no liver disease and CF participants with cirrhosis were not significantly different compared to mean values calculated using a 3T scanner, as shown in Table 3. Pairwise comparisons between groups in both the 1.5T and 3T cohort are shown in Table 4.

**Table 3:** Mean liver MRI ECV according to group using a 1.5T and a 3T scanner.

	Healthy volunteers	CF with no liver disease	CF with cirrhosis
**1.5T Cohort**	0.269 (0.048)	0.310 (0.037)	0.362 (0.063)
**3T Cohort**	0.316 (0.058)	0.297 (0.034)	0.388 (0.067)
	*p* = 0.14	*p* = 0.54	*p* = 0.65

**Table 4:** Pairwise statistical comparisons between groups using a 1.5T and a 3T scanner.

	Healthy volunteers vs CF with no liver disease	Healthy volunteers vs CF with cirrhosis	CF with no liver disease vs CF with cirrhosis
**1.5T Cohort**	*p* = 0.11	*p* = 0.02	*p* = 0.18
**3T Cohort**	*p* = 0.55	*p* = 0.09	*p* = 0.05

**Fig 3:**
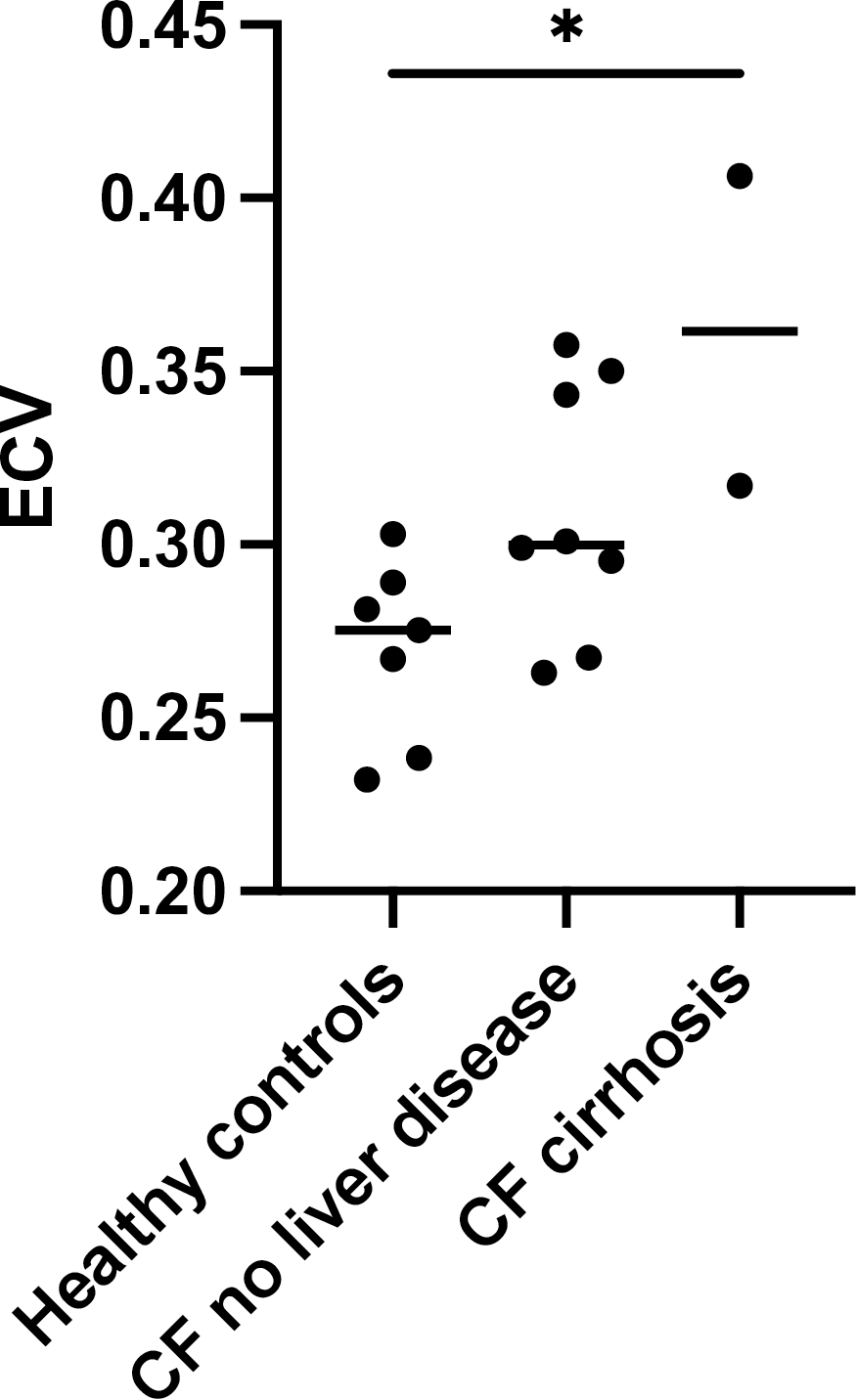
MRI ECV values according to group on 1.5T MRI scanner.

In this cohort, liver MRI ECV of healthy volunteers (0.269, SD 0.048) was significantly lower than that of CF participants with cirrhosis (0.362, SD 0.063) *p* =  0.02. Although MRI ECV of healthy volunteers (0.269, SD 0.048) was lower than CF participants with no liver disease (0.310, SD 0.037) this difference was not significant, *p* =  0.11. CF participants with cirrhosis had a higher MRI ECV (0.362, SD 0.063) compared to CF participants with no liver disease (0.310, SD 0.037) which was not significant, *p* =  0.18. These results are shown in Fig 3.

## Discussion

This pilot study demonstrates the feasibility of using standard MRI equipment and a simple protocol to quantify ECV in the liver of PwCF. This may be of diagnostic utility in those with advanced liver disease, as PwCF with cirrhosis had higher MRI ECV compared to healthy controls or PwCF with no liver disease.

Although sample sizes were small, mean ECV values for CF participants with cirrhosis were similar between 1.5T and 3T machines (0.362 and 0.388 respectively) as well as mean ECV values for CF participants with no liver disease (0.310 vs 0.297). This may suggest potential applicability across scanners of different field strength, although in this small study there was less concordance between mean ECV values for healthy controls measured by different field strength scanners (0.269 vs 0.316). Furthermore, the difference between ECV values in CF participants with no liver disease and CF participants with cirrhosis was not significant when measured using a 1.5T machine. Unlike other technologies to assess liver fibrosis such as MRE, MRI ECV utilises standard MR equipment that is commonplace in most advanced healthcare systems and can be performed using a protocol that does not add any additional time to that of a standard liver MRI.

In addition to showing feasibility, we were able to demonstrate a difference in MRI ECV between those with and without advanced CFLD, suggesting potential utility as a diagnostic tool in CFLD. International guidance recommends that CFLD should be classed according to severity [23], however current diagnostic tools are limited as ultrasound assessment can be subjective and there are concerns about the reliability of non-invasive LSM and liver biopsy given the focal nature of disease. Using a dual pass approach has been shown to improve the diagnostic accuracy of liver biopsy, but due to its invasive nature has not become standard of care [26]. Although MRI ECV may be able to measure liver fibrosis in a larger liver volume compared to other techniques such as FibroScan, this difference is modest (considering an approximate standard liver volume of 1200 cm^3^). However, MRI ECV is non-invasive and may have the potential additional benefit of being able to selectively measure liver fibrosis in those with radiological evidence of fibrosis or nodularity.

In this study we deliberately identified PwCF with firm radiological evidence of cirrhosis compared to those who did not meet diagnostic criteria for CFLD. This ensured our cohorts had low chance of mis-categorisation; a risk when a gold-standard is not available to help distinguish borderline cases. Our results demonstrated a higher MRI ECV in CF participants with cirrhosis compared to CF participants without liver disease, which was also seen in those where MRI ECV was calculated using a 1.5T scanner (though the small number of CF participants with cirrhosis in this cohort limit statistical interpretation). This suggests a potential role for MRI ECV as a diagnostic tool to detect liver fibrosis in PwCF, however in practice the clinical spectrum of CFLD is heterogeneous and people with CFLD can often present with a predominant phenotype of portal hypertension, both with and without cirrhosis [3]. The applicability of MRI ECV in individuals with non-cirrhotic portal hypertension is therefore unclear.

The main limitation of this proof-of-concept study is the small sample size, making it difficult to ascertain the statistical significance of these findings. Further study in larger cohorts is warranted to confirm if this technology can be used in PwCF to aid diagnosis of advanced liver fibrosis and cirrhosis. Based on results from this study, a sample size of approximately 42 is estimated to provide results with a power of 90%. In addition, our results are only applicable to an adult population and further work may be needed to assess the feasibility of liver MRI ECV in younger age groups, when CFLD is often diagnosed.

In summary, this proof-of-concept pilot study suggests that it is feasible to assess liver ECV using a 3T Siemens MR scanner in people with CF, with a trend towards a higher ECV in those with advanced CFLD compared to CF participants without liver disease. Furthermore, we have shown similar results on a more readily available 1.5T Siemens MR scanner, suggesting broader applicability. Given the limitations of current diagnostic and monitoring strategies for CFLD, further investigation of the utility of MRI ECV in this population is warranted.

## Supporting information

S1 Data Fully anonymized data file. (XLXS)
